# PCycDB: a comprehensive and accurate database for fast analysis of phosphorus cycling genes

**DOI:** 10.1186/s40168-022-01292-1

**Published:** 2022-07-04

**Authors:** Jiaxiong Zeng, Qichao Tu, Xiaoli Yu, Lu Qian, Cheng Wang, Longfei Shu, Fei Liu, Shengwei Liu, Zhijian Huang, Jianguo He, Qingyun Yan, Zhili He

**Affiliations:** 1 Environmental Microbiomics Research Center, School of Environmental Science and Engineering, Southern Marine Science and Engineering Guangdong Laboratory (Zhuhai), State Key Laboratory of Biocontrol, Sun Yat-sen University, Guangzhou, 510006 China; 2 Institute of Marine Science and Technology, Shandong University, Qingdao, 266237 China; 3 College of Agronomy, Hunan Agricultural University, Changsha, 410128 China

**Keywords:** Phosphorus cycling gene/microorganism, Database, Accuracy, Comprehensiveness, Metagenome sequencing data

## Abstract

**Background:**

Phosphorus (P) is one of the most essential macronutrients on the planet, and microorganisms (including bacteria and archaea) play a key role in P cycling in all living things and ecosystems. However, our comprehensive understanding of key P cycling genes (PCGs) and microorganisms (PCMs) as well as their ecological functions remains elusive even with the rapid advancement of metagenome sequencing technologies. One of major challenges is a lack of a comprehensive and accurately annotated P cycling functional gene database.

**Results:**

In this study, we constructed a well-curated P cycling database (PCycDB) covering 139 gene families and 10 P metabolic processes, including several previously ignored PCGs such as *pafA* encoding phosphate-insensitive phosphatase, *ptxABCD* (phosphite-related genes), and novel *aepXVWPS* genes for 2-aminoethylphosphonate transporters. We achieved an annotation accuracy, positive predictive value (PPV), sensitivity, specificity, and negative predictive value (NPV) of 99.8%, 96.1%, 99.9%, 99.8%, and 99.9%, respectively, for simulated gene datasets. Compared to other orthology databases, PCycDB is more accurate, more comprehensive, and faster to profile the PCGs. We used PCycDB to analyze P cycling microbial communities from representative natural and engineered environments and showed that PCycDB could apply to different environments.

**Conclusions:**

We demonstrate that PCycDB is a powerful tool for advancing our understanding of microbially driven P cycling in the environment with high coverage, high accuracy, and rapid analysis of metagenome sequencing data. The PCycDB is available at https://github.com/ZengJiaxiong/Phosphorus-cycling-database.

Video Abstract

**Supplementary Information:**

The online version contains supplementary material available at 10.1186/s40168-022-01292-1.

## Background

Phosphorus (P) is an essential nutrient for energy metabolism, genetic materials, and cell structures of all biota [1]. Unlike nitrogen (N), which has the volatile form of N (e.g., N_2_, N_2_O), the atmosphere does not supply soluble P [2]. Therefore, P is the second most limiting nutrient because the primary source of P relies on weathering of rocks in natural ecosystems [3]. P limitation could be alleviated by applying P fertilizers in the agroecosystem [4], but excessive P applications can cause serious water pollution and eutrophication. Also, bacteria and archaea (hereafter microorganisms) play important roles in maintaining and regulating the P status through inorganic P (Pi) solubilization and organic P (Po) mineralization and thus increase nutrient acquisition by plants [5, 6]. For example, phosphate solubilizing microorganisms could release orthophosphate from organic materials by secreting hydrolytic enzymes and organic acids, thus increasing available P around the crop rhizosphere [6]. However, comprehensive understanding of P cycling genes (PCGs) and microorganisms (PCMs) remains unclear.

PCGs are generally classified into “extracellular” and “intracellular genes.” The former is further clustered into three groups: Pi solubilization and Po mineralization genes (e.g., *gcd*, *phy*, *phoD*, and *phnJ*), transporter genes (e.g., *pstS*, *ugpQ*), and P starvation regulation genes (e.g., *phoB*, *phoR*) [1, 5, 7]. PhoB activated by phosphorylated-PhoR upregulates the expression of transporters (e.g., PstSCAB) and phosphatases (e.g., PhoD, PhoA) to utilize P under deficiency [8], but the SenX3-RegX3 two-component system instead of PhoB-PhoR responds to P starvation in mycobacteria [9]. Except for C-P lyase genes (e.g., *phnHIJKL*), phosphonates containing about 30% of high-molecular-weight dissolved organic phosphorus (DOP) in the marine environment could be used by microorganisms through various processes mediated by transaminase gene (*phnW*), hydrolase gene (*phnX*), phosphonate breakdown factor A (*pbfA*), dehydrogenase gene (*phny*), dioxygenase gene (*phnY*), and oxygenase gene (*phnZ*) [10–14]. Strikingly, PCGs involved in microbial metabolic processes are defined as “intracellular genes” and are often excluded because they are not considered as a part of natural P turnover, and/or they do not typically participate in P cycling. However, these “intracellular genes” indeed mediate the biosynthesis of key phosphorus compounds. For example, α-D-ribose-1-diphosphate-5P (PRPP), a key phosphonate compound in the nucleotide biosynthesis (i.e., purine and pyrimidine), could be synthesized by ribose 1,5-bisphosphokinase (*phnN*, once considered as an “extracellular gene”) and ribose-phosphate pyrophosphokinase encoded by *prsA*, which was excluded as an “intracellular gene” [15]. Moreover, phosphonoacetaldehyde is the central phosphonate compound for organophosphonate assembly such as 2-aminoethylphosphonate (2AEP), phosphonoacetate, and methylphosphonate [11]. Thus, it is pivotal to expand PCGs beyond the current-defined “extracellular genes” for mechanistic understanding of P cycling processes and cellular P metabolisms in the environment.

Isolation of phosphate cycling bacterial strains and sequencing of functional and 16S rRNA gene amplicons have provided new insights for microbially driven P cycling and possible mechanisms [16–18]. However, high proportions of microorganisms in diverse environments remain uncultured [19]. Also, it has been reported that about 20% of bacteria would be undetectable using currently available primers due to well-recognized biases [20]. Recently, metagenome sequencing analysis has proven to be a powerful method for understanding the microbially driven biogeochemical cycling (e.g., phosphorus, nitrogen, carbon, sulfur, and metals) in natural and engineered environments [21–24]. However, our understanding of P cycling microbial communities and their ecosystem functioning is still limited [25], and one of the major reasons is the lack of a comprehensive and accurately annotated database for analyzing PCGs and PCMs.

A comprehensive and accurate database is crucial for analyzing specific functional processes, pathways, and genes such as *nifHK* for N_2_ fixation, *asrABC* for sulfur reduction, *pmoABC* for methane oxidation, *mcr-1* for antibiotic resistance, and *intI1* for mobile genetic elements as well as their associated microbial groups [26–30]. So far, a few orthology databases are available to decipher the functional genes/pathways from metagenome sequencing data [31–35]. These available databases contain various types of genes involved in many biogeochemical cycles, but still face great challenges, such as the low coverage of functional genes/pathways, inaccurate annotations, exclusion of newly discovered genes, and long run-time. A recent study developed a pipeline for analyzing phosphatases in soil metagenomes using BLASTP search coupled with hidden Markov modeling, but this method needs manually curation [36]. The recently developed specific “small databases” such as NCycDB and SCycDB have been used to profile nitrogen and sulfur cycling microbial communities with high coverage, accuracy, and short run-time [26, 28]. As more and more qPCR and metagenomic analyses detect PCGs in different environments [1, 5, 37], the recovery of PCG diversity from metagenome sequencing data has become a demanding task. Hence, it is necessary to develop a comprehensive, well-annotated, and well-validated database to fast profile P cycling microbial communities in the environment.

Here, we aimed to develop a comprehensive and accurate P cycling functional gene database to accurately and rapidly analyze P cycling genes from the environment through metagenome sequencing data. We selected currently known 139 gene families from 10 P metabolic processes to construct a curated P cycling database (PCycDB), which was integrated with four publicly available orthology databases and the NCBI RefSeq database. We applied criteria (e.g., identity, hit length) to filter sequence alignment results to reduce false positives. Additionally, we applied PCycDB to analyze the distribution of PCGs in seven habitats including deep sea, eutrophic lake, mangrove, mariculture, surface ocean, permafrost, and wastewater treatment plant (WWTP). We demonstrate that PCycDB provides a new tool for comprehensive, accurate, and rapid analysis of P cycling microbial communities. Furthermore, PCycDB could also be used to annotate PCGs with the sequences obtained from other platforms (e.g., MiSeq, MinION).

## Methods

### P cycling database construction

**Fig. 1:**
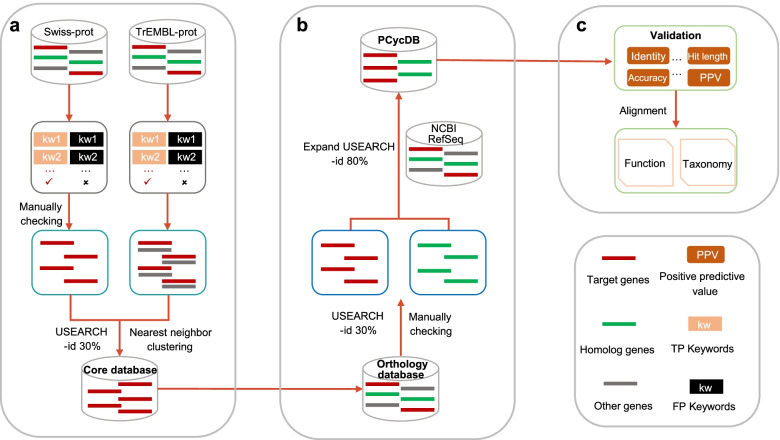
The technical flow for PCycDB construction. TP, true positives; FP, false positives

A modified method (Fig. 1) was developed to construct the PCycDB by integrating the UniProt, arCOG, COG, eggNOG, KEGG, and NCBI archaeal and bacterial RefSeq databases [26, 28]. The initial collection of PCG families (e.g., *pafA*, *gcd*, *pstSCAB*, *phoA*) and function descriptions were retrieved based on previous literatures [1, 5, 7, 10, 12, 38–46]. KEGG is a comprehensive database resource to analyze gene functions and utilities of the biological system [32]. Therefore, numerous phosphorus metabolism pathways (e.g., pyruvate metabolism, pentose phosphate pathway) in the KEGG database were referred to obtain PCG families for microbial metabolic processes (e.g., *pps*, *deoB*, *purD*) and function descriptions (Additional file 1: Table S1).

Candidate PCGs were first extracted from the Swiss-Prot database, which has been manually annotated, by keyword search against their gene names or function descriptions (Fig. 1a, Additional file 1: Table S1) [47]. For those gene families whose sequences were not included in the Swiss-Prot (e.g., *pbfA*, *phoX*, and *aepVXWPS*), we manually retrieved them from IMG database according to the literatures [12, 45, 46]. Also, we included two novel *phoA* genes (Fjoh_3187 and Fjoh_3249) identified in *Flavobacterium johnsoniae* DSM2064 [45]. Candidate PCGs were then carefully checked based on their annotation to ensure the reliability of the developed database. In addition, for those PCG sequences extracted from the TrEMBL database but without full manual annotations, they were merged with candidate sequences by a self-versus-self alignment using USEARCH v.11.0 with a 30% global identity, followed by a nearest neighbor clustering procedure to generate the core database for PCG families [26, 48].

The core database was expanded against four orthology databases including arCOG (ftp://ftp.ncbi.nih.gov/pub/wolf/COGs/arCOG/, version ar14), COG (ftp://ftp.ncbi.nih.gov/pub/COG/COG2020/data/, version COG2020), eggNOG (http://eggnog5.embl.de/download/eggnog_5.0/, version 5.0), and KEGG (http://www.genome.jp/kegg/, downloaded on Oct. 2, 2021) using USEARCH v.11.0 with a global identity of 30%. The representative sequences and homologues of PCGs were identified, extracted, and integrated by manually checking their annotation results from the alignment table (Fig. 1b). Because the representative sequences of some gene families (e.g., *gnd*, *ppk*, *pstB*, *purFDNTL*) retrieved from eggNOG and KEGG have an average identity over 95% against the core database, these sequences were clustered by CD-HIT at 95% identity to make PCycDB more compactable [49]. The NCBI RefSeq database (ftp://ftp.ncbi.nlm.nih.gov/refseq/release/, downloaded on Oct. 2, 2021) was employed to improve the comprehensiveness and integrality by searching against the developed PCycDB using USEARCH v.11.0 with an 80% global identity [27]. It should be noted that a strict cutoff (i.e., 80%) was applied in this step because of a large number of sequences in the NCBI RefSeq database. All amino acid representative sequences and nontarget homologues were de-duplicated and clustered by CD-HIT at 100% identity [49]. Finally, all representative sequences and homologues were selected to construct PCycDB.

### Simulated gene dataset

As NCycDB is a manually curated database with N cycling gene families [26], those N cycling gene sequences were selected as true negatives for PCycDB validation. Then, a simulated gene dataset (Additional file 2: Simulated_gene_datase.fasta) containing 139 PCG families (12,972 sequences) and 68 N cycling gene families (219,091 sequences) was constructed and compared against PCycDB using DIAMOND with an *e*-value of ≤ 10^−5^ to estimate the accuracy of PCycDB (Fig. 1c). Although homologous sequences of NCycDB were excluded to increase the credibility, it should be noted that some genes have multiple functions. For example, *phoR* encoding a phosphate regulon sensor protein is defined as a benzalkonium chloride resistance gene in the BacMet database [50]. Thus, a few N cycling genes might be also considered as PCGs, resulting in false positives. To evaluate the accuracy of PCycDB, we calculated accuracy, positive predictive value (PPV), specificity, sensitivity, and negative predictive value (NPV) based on the following equations:

1 $$Accuracy=\frac{True\ Positives+ True\ Negatives}{True\ Positives+ False\ Positives+ True\ Negatives+ False\ Negatives}$$

2 $$Positive\ predict\ value=\frac{True\ Positive s}{True\ Positive s+ False\ Positive s}$$

3 $$Specificity=\frac{True\ Negatives}{True\ Negatives+ False\ Positives}$$

4 $$Sensitivity=\frac{True\ Positives}{True\ Positives+ False\ Negatives}$$

5 $$Negative\ predict\ value=\frac{True\ Negative s}{True\ Negative s+ False\ Negative s}$$

### A genome sequence dataset from a mock community

Considering that the whole genome sequencing and metagenome binning have been widely used to study the metabolic pathway in an individual microorganism, a mock microbial community (Additional file 3: Mock_community.fasta) containing 50 bacterial genomes was constructed to further validate the accuracy of PCycDB (Fig. 1c). The protein sequences (.fasta file) and genome annotations (.gff file) of each genome were randomly retrieved from the NCBI genome or assembly database. The detection ratio was calculated by dividing the number of PCG families predicted using PCycDB by the number of those described by the NCBI genome or assembly database. Those genomes with detection ratio > 1.0, = 1.0, or < 1.0 were defined as overestimated, exactly estimated, and underestimated, respectively. The simulated gene and mock community sequence datasets were searched against PCycDB using DIAMOND with an *e*-value ≤ 10^−5^.

### Metagenome sequencing datasets

To test PCycDB applications for various environments, the developed PCycDB was used to analyze PCGs from seven habitats including deep sea (*n* = 6), eutrophic lake (*n* = 5), mangrove (*n* = 8), mariculture (*n* = 13), surface ocean (*n* = 6), permafrost (*n* = 9), and WWTP (*n* = 8). Metagenome sequencing datasets were collected from sequence read archive (SRA) in NCBI. To prevent fluctuations produced by different sequencing strategies, only metagenome data sequenced by the Illumina HiSeq platform with paired-end sequencing were selected (Additional file 1: Table S2).

### Function and taxonomy annotation

Each metagenome was quality trimmed using sickle with a paired-end mode and a minimal quality of 20 [51]. The high-quality reads were assembled into contigs via *de Bruijn* graph with a multiple *k*-mer size (parament: --k-list 21, 29, 39, 59, 79, 99, 119, 141) strategy using MEGAHIT [52]. The open reading frames (ORFs) were predicted using Prodigal v2.6.3 [53] and annotated by searching against arCOG, COG, eggNOG, KEGG, and PCycDB using DIAMOND with an *e*-value of ≤ 10^−5^ and the same computational thread (option: −p 20). Only the alignment results aligned to PCycDB were filtered with an identity ≥ 30.0% and hit length ≥ 25 amino acids (aa). The ORF abundance (coverage) was calculated using the following equation:

6 $$Coverage=\sum_1^n\frac{N\times l/L}{S}$$

where *N* is the number of reads mapped to predicted ORFs, *L* is the sequence length of a target ORF, *n* is the number of predicted ORFs, *l* is the length of Illumina sequencing reads, and *S* is the sequencing data size (Gb) [54]. Meanwhile, those *pafA* ORFs were extracted to profile the taxonomical composition based on the BLASTX search against the NCBI nonredundant database.

### Statistical analysis

All analyses were performed using R 4.0.5 if not specified. The most important filtering paraments for increasing the accuracy of PCycDB were determined using random forest analysis based on the alignment result. Seventy percent of alignment results were used to train the fit model, while the remaining was used to validate the accuracy of model. The two-tailed analysis of variance (ANOVA) was used to calculate the significant difference of detected gene families, run-time, and PCG coverage among seven habitats or different databases. The resulting *P*-values were adjusted by the Tukey’s multiple comparisons test using the GraphPad software (Version Prism 8.0.1, California, USA). The enrichment of PCGs within a habitat was tested by Fisher’s exact test with the *P*-value adjusted by Bonferroni correction. A nonmetric multidimensional scaling plot (NMDS) based on the Bray–Curtis distances was performed to reveal the beta diversity of PCGs. The significant difference of PCGs among different habitats was performed using multi-response permutational procedure (MRPP) and analysis of similarity (ANOSIM) tests.

## Results

### Gene families and metabolic processes in PCycDB

**Fig. 2:**
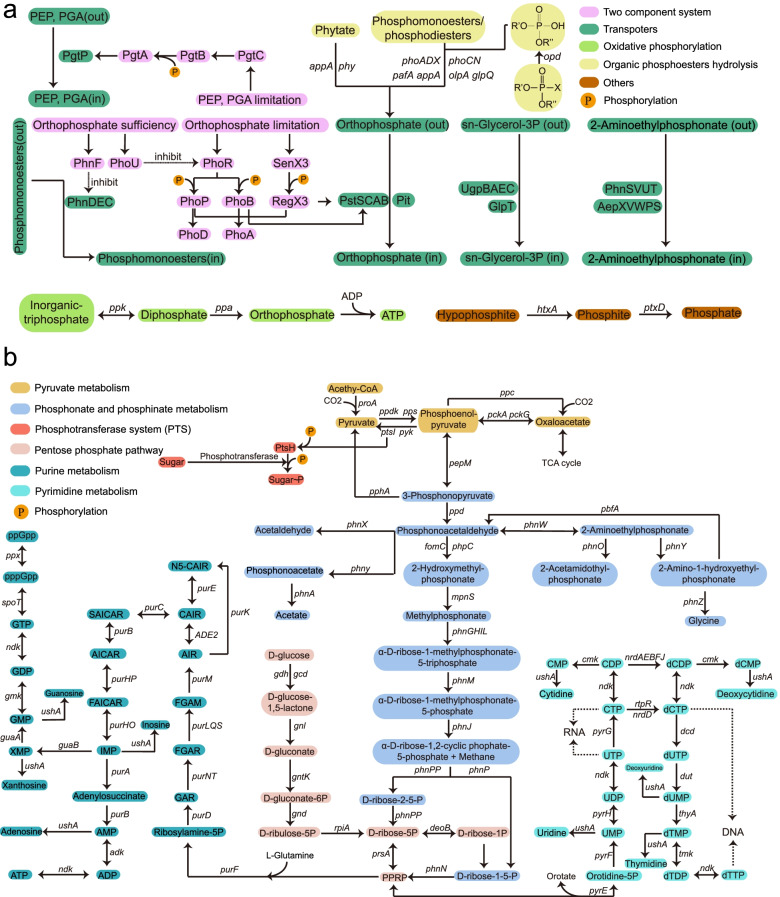
An outline of key phosphorus metabolic processes. **a** Two-component system, oxidative phosphorylation, transporters, and organic phosphoester hydrolysis. **b** Pyruvate metabolism, phosphonate and phosphinate metabolism, phosphotransferase system (PTS), pentose phosphate pathway, purine metabolism, and pyrimidine metabolism. X may be O, F, C, or S; R is any alkyl group. PEP, phosphoenolpyruvate; PGA, including 2-phosphoglycerate and 3-phosphoglycerate; PRPP, α-D-ribose-1-diphosphate-5P; GAR, 5′-phosphoribosylglycinamide; FGAR, 5′-phosphoribosyl-N-formylglycinamide; FGAM, 2-(formamido)-N1-(5′-phosphoribosyl) acetamidine; AIR, aminiimidazole ribotide; CAIR, 1-(5′-phospho-D-ribosyl)-5-amino-4-imidazolecarboxylate; N5-CAIR, 5-carboxyamino-1-(5-phospho-D-ribosyl)imidazole; SAICAR, 1-(5′-phospho-D-ribosyl)-5-amino-4-(N-succinocarboxamide)-imidazole; AICAR, 1-(5′-phosphoribosyl)-5-amino-4-imidazolecarboxamide; FAICAR, 1-(5′-phosphoribosyl)-5-formamido-4-imidazolecarboxamide

We identified 139 key PCG families based on 863,513 representative sequences and 320,183 homologues covering 10 phosphorus cycling processes (Additional file 1: Table S1). Two-component system, oxidative phosphorylation, transporters, and organic phosphoester hydrolysis are major processes for microbes to regulate, transport, and uptake P sources from the environment (Fig. 2a), and pyruvate, pentose phosphate, phosphonate and phosphinate, purine, and pyrimidine metabolisms are responsible for cellular P metabolic processes to synthesize organic P compounds (Fig. 2b).

#### Two-component system

A total of nine gene families with 50,866 representative sequences and 13,780 homologues are retrieved for two-component system, including *phoU*, *phoR*, *phoB*, *phoP*, *SenX3*, *RegX3*, *pgtC*, *pgtB*, and *pgtA*. These regulons are activated to modulate the expression of transporter genes (e.g., *pstSCAB*, *pgtP*) and phosphatase genes (e.g., *phoA*, *phoD*) under P depletion.

#### Transporters

Twenty-eight gene families including *pgtP*, *pstSCAB*, *pit*, *htxB*, *ptxABC*, *phnD_phosphite*, *phnDEC*, *ugpBAEC*, *phnSVUT*, *glpT*, and *aepXVWPS* are recruited for transporters. A total of 115,660 sequences and 114,711 homologous orthology groups are collected. The orthophosphate outside the membrane is transported into the cell by permease proteins encoded by *pstSCAB* and *pit*. The hypophosphite and phosphite could be transported into cell by HtxB and PtxABC protein, respectively. Phosphoenolpyruvate (PEP) and phosphoglycerate (PGA) enter the cell by binding to a phosphoglycerate transporter protein (PgtP), while sn-glycerol-3P is transported by proteins encoded by *ugpBAEC*. The ATP-binding cassette transporters including PhnDEC, PhnSVUT, and AepXVWPS are responsible for 2-aminoethylphosphonate (2AEP) transport.

#### Organic phosphoester hydrolysis

Thirteen gene families with 15,902 sequences and 1022 homologues are collected in the organic phosphoester hydrolysis process. Among them, *phoA*, *phoD*, and *phoX* code for alkaline phosphatases, and *phoN*, *aphA*, *phoC*, and *olpA* encode acid phosphatases. The *opd* gene encodes phosphotriesterase, and *phy* and *appA* code for phytases. The *pafA* gene encodes Pi-insensitive phosphomonoesterase, and *ugpQ* and *glpQ* code for cytoplasmic glycerophosphoryl diester phosphodiesterase and periplasmic glycerophosphoryl diester phosphodiesterase, respectively.

#### Pyruvate metabolism

Six gene families, including *pps*, *ppdK*, *pyk*, *pckG*, *ppc*, and *pckA*, are involved in pyruvate metabolism. Phosphoenolpyruvate is synthesized by dikinases (i.e., *pps*, *ppdK*) and carboxykinases (i.e., *pckG*, *pckA*). A total of 42,872 representative sequences and 7824 homologues are included in this metabolic process.

#### Pentose phosphate pathway

The pentose phosphate pathway contains eight gene families including *gdh*, *gcd*, *gnl*, *gntK*, *gnd*, *rpiA*, *prsA*, and *deoB* with 49,974 representative sequences and 22,752 homologous sequences. PRPP can be synthesized through this pathway for nucleotide biosynthesis.

#### Phosphotransferase system

Gene families including *ptsI* and *ptsH* are included in the phosphotransferase system. The *ptsI* gene codes for phosphoenolpyruvate-protein phosphotransferase, and *ptsH* encodes a phosphocarrier protein. A total of 11,192 sequences and 2539 homologous orthology groups are collected.

#### Oxidative phosphorylation

Two gene families including *ppk* and *ppa* are recruited in this metabolic process with a total of 32,190 representative sequences and 5028 homologous sequences.

#### Phosphonate and phosphinate metabolism

A total of 24 gene families including *pepM*, *pphA*, *ppd*, *fomC*, *phpC*, *mpnS*, *phnGHIJKLMNOPWXYZ*, *phny*, *pbfA*, and *phnPP* are involved with a total of 31,285 sequences and 20,862 homologues. Three important metabolic pathways including *phnW*-*phnX*, *phnW*-*phny*, and *phnY*-*phnZ* are responsible for the degradation of 2AEP to produce acetaldehyde, phosphonoacetate, and glycine, respectively.

#### Purine metabolism

Purine metabolism contains 25 gene families including *ADE2*, *adk*, *gmk*, *ushA*, *guaAB*, *ndk*, *ppx*, *purABCDEFHKLMNOPQST*, and *spoT* with a total of 333,930 representative sequences and 79,528 homologous sequences. These gene families are responsible for the biosynthesis of ATP and GTP.

#### Pyrimidine metabolism

Pyrimidine metabolism is composed of 18 gene families including *dcd*, *dut*, *cmk*, *ushA*, *ndk*, *nrdABDEFJ*, *pyrEFHG*, *rtpR*, *thyA*, and *tmk* with a total of 191,825 sequences and 52,535 homologous orthology groups. These gene families are responsible for the biosynthesis of TTP and CTP as well as DNA and RNA. The *ndk* gene encoding a nucleoside-diphosphate kinase is included in both purine and pyrimidine metabolisms because it mediates the production of ATP, GTP, CTP, TTP, and UTP during nucleotide metabolism (Additional file 1: Table S1). The *ushA* gene encoding 5′-nucleotidase has an important function in nucleotide (e.g., AMP, GMP, and IMP) salvage.

#### Others

Six gene families including *htxA*, *ptxD*, *lysR*, *phnR*, *phnF*, and *phoH* are also included in PCycDB. The *phnR* gene is the regulator for induction of *phnA*, and the LysR protein activates the transcription of *phnWX* operon. The hypophosphite and phosphite could be oxidized by HtxA and PtxD, respectively. A total of 7520 sequences and 642 homologues are identified.

### Validation of PCycDB with a simulated gene dataset

**Fig. 3:**
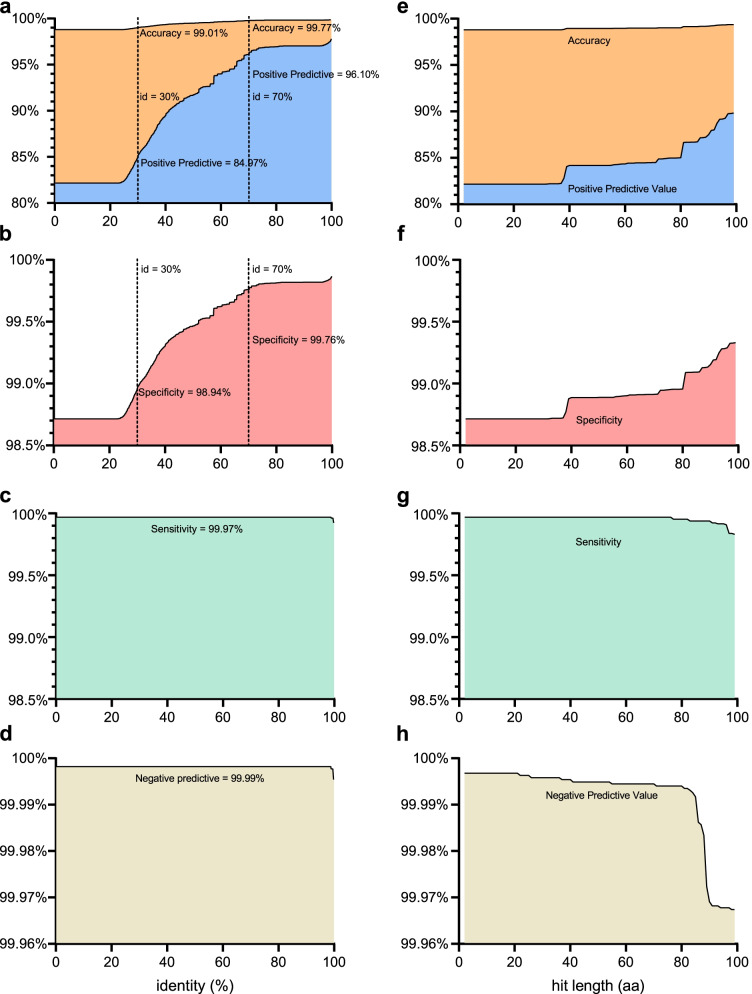
The accuracy of PCycDB against identity and hit length. The positive predictive value (PPV, **a**, **e**), sensitivity (**b**, **f**), specificity (**c**, **g**), and negative predictive value (NPV, **d**, **h**) were recorded along with the identity varied from 0.2 to 99.9% with a step by 0.1%, and the hit length ranged from 2 to 99 amino acids with a step of one. Left dash line represents a 30% identity cutoff, and right dash line means a 70% identity cutoff

We first used a simulated gene dataset coupling with random forest analysis to evaluate the accuracy of PCycDB. The fit model with an accuracy of 98.6% suggested that the identity and hit length were the two most important factors to discriminate true negatives from false positives (Additional file 1: Table S3, S4). Positive predictive value (PPV) and specificity followed an S-shape curve and remarkably increased with identity (from 23.0 to 82.0%), indicating that false positives could be efficiently removed as identity increased (Fig. 3a and b). For example, when accuracy was 99.0% with a 30.0% identity cutoff, PPV and specificity were 85.0% and 98.9%, respectively, and no reduction of sensitivity or negative predict value (NPV) was observed when the identity increased from 0.2 to 95.9% (Fig. 3c and d). Similarly, PPV and specificity increased with hit length (Fig. 3e and f), but sensitivity and NPV dramatically decreased when the hit length was ≥ 80 aa (Fig. 3g and h). These findings suggested that hit length was not an effective filtering factor to increase the accuracy of PCycDB; thus, the cutoff of hit length was empirically set to 25 aa [55]. With the above criteria (i.e., 30.0% identity and 25 aa), the detection ratio of specific PCG was 99.9 ± 0.6% (Additional file 1: Table S5), that is, all the known PCGs were sensitively detected by PCycDB.

### Validation of PCycDB with a mock community

**Fig. 4:**
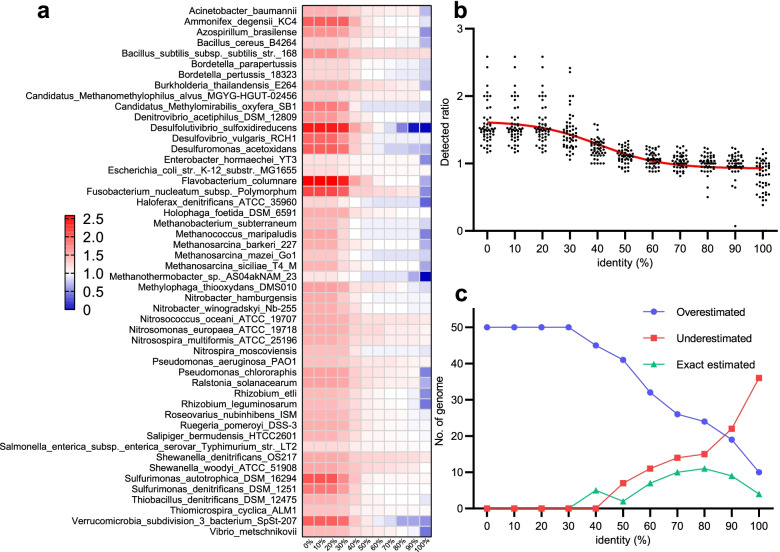
The accuracy of PCycDB validated by a mock community. **a** Heatmap showed the detection ratio of each genome involved in the mock community as identity increased, and white represents the detection ratio of 1.0. **b** The discrete tendency of detection ratios. **c** The number of over-, under-, or exact-estimated genomes at different identity cutoffs

We further used a mock community containing 50 microbial genomes to validate PCycDB and found that all genomes were overestimated with an identity cutoff of 30.0% (Fig. 4a). The detection ratio varied from 1.05 (*Methanothermobacter* sp. AS04akNAM 23) to 2.42 (*Flavobacterium columnare*) with an average of 1.47 ± 0.28. The results were consistent with the simulated gene dataset, indicating the high false positives at a relatively low identity cutoff for genomes (i.e., 30.0%, Fig. 4b). The number of over-, exact-, and underestimated genomes was 19, 9, and 22, respectively, at the 90.0% identity. Unlike PPV calculated with the simulated gene dataset, which showed a plateau phase, the detection ratio of some bacterial genomes (e.g., *Desulfolutivibrio sulfoxidireducens*) was substantially reduced at a high identity cutoff, causing false negatives (Additional file 1: Table S6). Interestingly, we observed an inverted V shape curve of exactly estimated genomes against identity (Fig. 4c). Of these 50 genomes, the number of exact estimated genomes reached a maximum at 80.0% identity (the turning point), with the detection ratios ranging from 0.81 (*Desulfuromonas acetoxidans*) to 1.15 (*Fusobacterium nucleatum* subsp. *polymorphum*). However, considering that the detection ratio was 1.03 (slightly higher than 1) and the standard deviation was relatively low (0.10), we believed that using an identity of 70% should be suitable for genome annotation. In this case, the number of over-, exact-, and underestimated genomes was 26, 14, and 10, respectively, while annotation accuracy, PPV, sensitivity, specificity, and NPV for simulated gene dataset were 99.8%, 96.1%, 99.9%, 99.8%, and 99.9%, respectively.

### Comparison of performance among different orthology databases

**Fig. 5:**
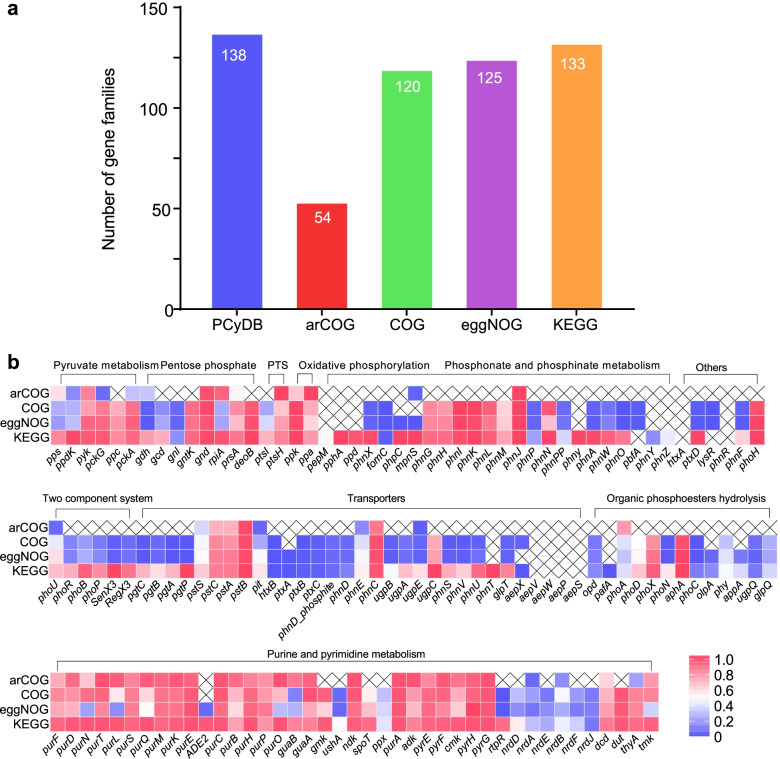
Comparison of performance among different orthology databases. **a** The number of PCG families detected in different databases. **b** Comparison of comprehensiveness of PCGs families in four public orthology databases. PCycDB was used as a reference (i.e., 100%) for the comparison. “×” indicates that this gene family is absent in corresponding database. The baseline was set to 0.5 and colored as white, that is, blue color represents the comprehensiveness less than 0.5

To evaluate the performance of PCycDB, we first compared the comprehensiveness of PCycDB with other publicly available orthology databases. In comparison with PCycDB with 139 PCGs families, arCOG, COG, eggNOG, and KEGG contain 54, 120, 125, and 133 PCG families, respectively (Fig. 5a), and they only have approximately half of representative sequences (Fig. 5b, blue cells). Especially, these orthology databases provide fewer representative sequences for acid and alkaline phosphatases (e.g., *phoC*, *phoN*, *phoX*) and phytases (e.g., *phy*, *appA*). Also, some key PCG families were still missing in the arCOG, COG, eggNOG, or KEGG databases, such as *ppd*, *htxA*, *aepVXWPS*, *phnZ*, and *phnR*. Second, the genes of *phoA*, *phoD*, *phoD*, and *pafA*, which are divergent PCG families, could be well phylogenetically separated in PCycDB (Additional file 4: Fig. S1). However, we also observed that *Flavobacterium* PhoX was phylogenetically distant from other *phoX* genes, while PafA and PhoD clusters were much closer, which are consistent with recent studies [45, 46]. Third, we compared the detected PCG families and run-time of metagenome sequencing datasets using those databases. Among them, PCycDB detected more PCG families with an average of 117.0 compared to arCOG (62.6), COG (91.5), eggNOG (89.2), and KEGG (91.2) databases (Additional file 4: Fig. S2a), and the run-time of PCycDB (201.3 s) was significantly (*P* < 0.05, ANOVA test) shorter than that of eggNOG (1246.7 s) and KEGG (762.2 s) databases (Additional file 4: Fig. S2b). Thus, compared to these existing orthology databases, the specific PCycDB achieves a more comprehensive, more accurate, and faster analysis of PCGs from metagenome sequencing datasets.

### Functional diversity revealed by PCycDB

**Fig. 6:**
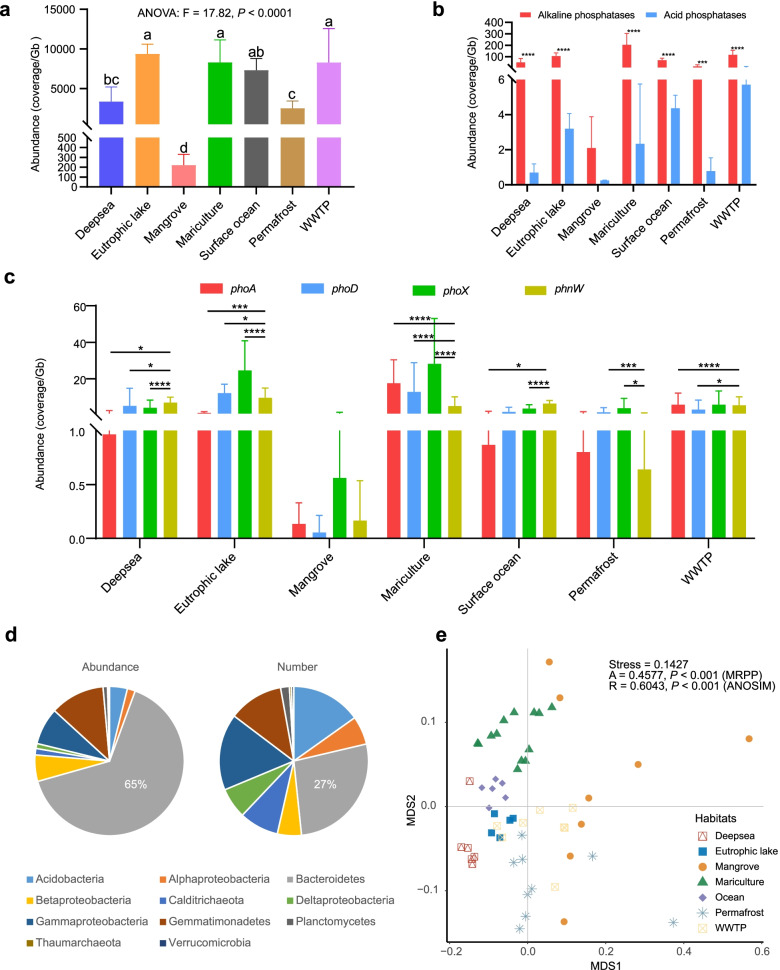
The composition and structure of PCGs in seven habitats (deep sea, *n* = 6; eutrophic lake, *n* = 5; mangrove, *n* = 8; mariculture, *n* = 13; surface ocean, *n* = 6; permafrost, *n* = 9; WWTP, *n* = 8). Bar plots showed the abundance of total PCGs (**a**), alkaline and acid phosphatases (**b**), and *phoA*, *phoD*, *phoX*, and *phnW* (**c**). Pie chart showed the taxonomical distribution of PafA (**d**). NMD analysis showed the beta-diversity of PCGs in seven habitats (**e**). The two-tailed analysis of variance (ANOVA) was used to calculate the significant difference of PCG abundance among seven habitats, and *P*-values were corrected by Tukey’s multiple comparison tests. The enrichment of the PCGs within a habitat was tested by Fisher’ exact test, with the *P*-value further adjusted using the Bonferroni correction. The significant difference of PCG patterns among different habitats was performed using multi-response permutational procedure (MRPP) and analysis of similarity (ANOSIM) tests. Different letters (“a,” “b,” “c,” or “d”) and asterisk represent the significant difference among these seven habitats. *p < 0.05; **p < 0.01; ***p < 0.001; ****p < 0.0001

We applied PCycDB to analyze the functional diversity of P cycling microbial communities from seven habitats, which represent typical habitats of natural and engineered ecosystems. The total PCGs in eutrophic lake, mariculture, and WWTP were more abundant than deep sea, permafrost, and mangrove (Fig. 6a). Purine and pyrimidine metabolism were the most abundant pathway in modulating P turnover, followed by transporters and two-component system, indicating a large P requirement in all habitats (Additional file 4: Fig. S3). However, the gene abundance for organic phosphoester hydrolase in all habitats was low. The abundance of *pstSCAB* genes was significantly (*P* < 0.01, Fisher’s exact test) higher than that of *phnDEC* genes excepted for deep sea and mangrove (Additional file 4: Fig. S4). The abundance of alkaline phosphatase genes (i.e., *phoA*, *phoD*, and *phoX*) was significantly higher than that of acid phosphatase genes in all habitats except for mangrove (Fig. 6b, *P* < 0.01, Fisher’s exact test). Interestingly, the abundance of *phnW* was significantly (*P* < 0.05, Fisher’s exact test) higher than that of *phoA*, *phoD*, and *phoX* in marine-associated deep sea and surface ocean (Fig. 6c). Although Bacteroidetes only constituted a low proportion number (27%) of *pafA* family, it accounted for 65% of the PafA abundance (Fig. 6d). NMDS plots revealed that the composition of PCGs was significantly (*P* < 0.001, MRPP and ANOSIM tests) different among seven habitats (Fig. 6e), suggesting a habitat-specific distribution of P cycling microbial communities.

## Discussion

Phosphorus, the eleventh most abundant element on Earth, is indispensable by all microbes for their pivotal metabolic functions [11]. It is important to rapidly decipher biogeochemical PCGs from metagenome sequencing data using an accurate database. In this study, we developed PCycDB with 139 gene families, identified key criteria (i.e., identity, hit length) for ensuring its annotation accuracy, and applied it to analyze PCGs from seven different habitats. The results demonstrate that PCycDB is a powerful tool for accurate, comprehensive and fast annotation of PCGs from the environment.

Compared to other databases, the PCycDB provides a more accurate annotation for metagenome sequencing data. First, the annotation of PCG families in PCycDB is more accurate. Some enzymes have equivocal annotations in the KEGG database. For example, the gene encoding phosphonoacetaldehyde dehydrogenase (defined as *phny* in this study, EC: 1.2.1) and the gene encoding 2-aminoethylphosphonate dioxygenase (defined as *phnY* in this study, EC: 1.14.11.46) were both named *phnY* in KEGG, resulting in ambiguity. In addition, the genes of *ugpQ* (cytoplasmic glycerophosphoryl diester phosphodiesterase) and *glpQ* (periplasmic glycerophosphoryl diester phosphodiesterase) have the same KO number (i.e., K00126). Second, the false positives are dramatically reduced by including 320,183 homologous sequences and implementing the best filtering parameters. To obtain the functional or taxonomic annotations from metagenome sequencing datasets, we generally search querying sequences against “large databases” such as NCBI nonredundant and/or KEGG databases using the default paraments as they include a certain set of sequences with different functions [21]. The bit score and *e*-value are the most commonly used filtering factors to promote the accuracy of functional annotations [5, 28]; however, false positives still remain with some functional gene databases [26]. For instance, assuming an ORF which was not *phoA* but had a 30.0% identity with *phoD* and a 60.0% identity with *phoA*, it would be annotated as *phoA* (false positive). This is the most frequent mistake when directly using the alignment results for downstream functional analysis. Including homologous sequences can reduce some false positives. In the above case, if that non-*phoA* ORF had a 70.0% identity with a homologous sequence, which had been added into the database, it would not be annotated as *phoA*. Although some non-PCGs can be excluded through the homologous method, false positives would still be present when non-PCG sequences had a higher identity with PCGs than homologues. Previously, the prediction of other function databases such as integrase, CARD, and BacMet databases is restricted to a high identity cutoff greater than 80% [27, 50, 56]. More recently, N cycling genes were annotated using NCycDB with an 85% identity threshold [26, 57]. However, false negatives can be increased at a high cutoff, that is, a considerable proportion of real functional genes would be removed [58]. Hence, it is crucial to set an appropriate cutoff to further reduce false positives and false negatives. In this study, Random forest analysis suggested that identity and hit length were the most powerful filtering factors to increase annotation accuracy. PPV, sensitivity, specificity, and NPV were all above 96.1% with a cutoff of 70.0% identity and 25-aa length, demonstrating the high accuracy of PCycDB to profile PCGs from metagenome sequencing datasets. The cutoff of hit length is empirically set to 25 aa because it is suitable for one to annotate short metagenome reads or predicted ORFs (e.g., 150 bp) without a significant increase of false negatives.

We noticed that certain genomes involved in the mock community had a detection ratio greater than 1.0 even with a 90.0% identity cutoff. This might be explained by the fact that annotations of these genomes from NCBI are obsolete. For example, the genome of *Bacillus subtilis* subsp. *subtilis* str. 168 with a detection ratio of 1.23 at a 90.0% identity was submitted in 2009, and since then, several new functional genes and pathways have been studied, greatly advancing our understanding of natural P cycling [10, 11, 43]. Moreover, the genus of *Bacillus* has long been known as P-solubilizing bacteria (PSB) capable of excellent phosphate solubilization and mineralization ability [4, 59]. The high detection ratio of PCGs in the mock community indicated that bacteria had more potential than previously recognized for mediating global P turnover. Therefore, we suggest that 30% identity and 25 aa are appropriate to profile PCGs from metagenome sequencing data because all known PCGs were detected with a small number of false positives (1.06%). Alternatively, one may use a stricter cutoff (i.e., 70.0% identity) to identify the PCGs from genomes and further reduce the false positives (< 0.25%).

We also demonstrate that PCycDB are more comprehensive compared to other databases. First, we include more PCG families in PCycDB. Previously, only some PCGs attributed to “extracellular enzymes” were investigated in forest soils, agroecosystems, and mined areas because these genes played an important role in respective research habitats [1, 5, 7]. Admittedly, phosphatases can be secreted outside the cell membrane by bacteria [1]. However, while the transporter system of *pstSCAB* and *phnDEC* complexes has been recognized as periplasmic binding proteins, there was no sufficient evidence to support that the *phnGHIJKLMNOP* genes encoding C-P lyases could also be released outside the membrane [60, 61]. Thus, the jargon of “extracellular genes” or “intracellular genes” should be carefully defined. Furthermore, it is important to uncover the metabolic mechanism of how microorganisms assimilate P into their biomass after acquiring orthophosphate. Second, we include more P cycling pathways in PCycDB. In addition to phosphoesters with C-O-P bonds, phosphonates that contain more stable C-P bonds consist of about one-third of total dissolved organic phosphorus (DOP) in marine environments [10]. 2AEP has been considered as the most abundant phosphonates in the ocean and could be transported by three transport systems, PhnDCE, PhnSTUV, and novel AepXVWSP [14, 38, 62]. It has been demonstrated that the genes of *aepXVW*, *aepS*, and *aepP* are Pi-insensitive, indicating the ecological role of marine and terrestrial bacteria capable of 2AEP catabolism [14]. In addition, three important metabolic pathways for the degradation of 2AEP are included in PCycDB, including *phnW*-*phnX*, *phnW*-*phny*, and *phnY*-*phnZ*. While *pstSCAB* and *phoD* are regulated by a two-component system PhoB-PhoR under P deficiency [63], the genes of *phnW* and *phnX* which cleave C-P bond of 2AEP are mediated by *lysR* [10], and phosphonoacetate hydrolase encoded by *phnA* is induced by another transcriptional regulator gene *phnR* [43]. A recent study reported that 2-amino-1-hydroxyethlyphosphonate (*R*-HEAP) could also be utilized by bacteria as a phosphorus source via a *pbfA*-*phnW*-*phny* pathway [12]. These genes are often excluded because they were not considered as participants in P cycling. Third, PCycDB have more representative sequences of PCGs. The arCOG database includes fewer types of PCGs families because it is designed for functional annotation of archaea [35]. However, COG, eggNOG, and KEGG have fewer representative sequences of PCGs especially for those involved in organic phosphoester hydrolysis processes; thus, the diversity/abundance of some PCGs could be underestimated. By including these “intracellular PCGs” which were usually neglected in previous studies and expanding the comprehensiveness of representative sequences, PCycDB facilitates the current insights into our understanding of microbial P cycling and metabolic mechanisms.

To understand the P cycling microbial communities in different environments, the profile of PCGs was deciphered using PCycDB. The results revealed that PCGs were widespread across different environments, indicating that P cycling is a common and important process in natural and engineered ecosystems. Meanwhile, we found that the composition of PCGs was variable among diverse habitats. The heterostructure of nutrient availability, temperature, moisture, and humic substances might play an important role in the intervention of unique PCG paradigm [64–66]. The high abundance of *pstSCAB* identified in this study was supported by the finding that the *pstSCAB* was a prominent transporter system for inorganic phosphorus uptake [67]. Phosphatases play a crucial role in acquiring P source from phosphoesters for microorganisms and mitigating eutrophication caused by P contamination [68, 69]. The genes encoding the alkaline phosphates were predominant in most habitats, indicating that the mineralization potential by hydrolyzing the C-O-P bonds was the main mechanism by which microorganisms acquired orthophosphate [7]. Intriguingly, *phnW* coding for 2AEP transaminase was prevalent across the environments examined. Similarly, a certain abundance of *phnW* was found in previous studies [1, 5]. These findings suggested that 2AEP was an important P source for microbes not only in marine environments but also in other habitats to meet their P requirements [11, 13]. The high abundance of *pafA* carried by Bacteroidetes indicates that Bacteroidetes lack most ATP-binding cassette transporters and need an additional way for organic molecules uptake [46]. These functional and taxonomic results evidence that PCycDB is a sensitive, accurate, and broad-spectrum database to analyze PCGs and PCMs in different environments.

## Conclusions

We developed an accurate, comprehensive, and well-curated P cycling functional gene database for metagenome sequencing data analysis with four orthology public databases and the NCBI RefSeq database integrated. Importantly, key genes encoding the intracellular P metabolic processes, Pi-insensitive phosphatase, and novel 2AEP transporters are included in the PCycDB, which should broaden our insights into microbially driven global biogeochemical P cycling. The accuracy is enhanced by including homologous sequences and using identity and hit length as effective filters. By applying the PCycDB to analyze P cycling microbial communities from seven habitats, we showed that PCycDB was widely applicable to accurately annotate PCGs from different environments. Thus, the constructed PCycDB is a powerful tool for rapidly analyzing P cycling microbial communities and their underlying mechanisms with high coverage and high accuracy.

## Supplementary Information

**Additional file 1: Table S1**. Summary of phosphorus gene families with representative sequences and orthology groups. **Table S2**. Public metagenomic datasets analyzed in this study. **Table S3**. The meandecreaseaccuracy of each filtering factor using randomForest package in R. **Table S4**. Confusion matrix result of Random Forest analysis using validation data. **Table S5**. Detection rate of each specific P cycling gene included in simulated gene dataset with an identity of 30%. **Table S6**. Predicted P cycling gene families.

**Additional file 2.**

**Additional file 3.**

**Additional file 4: Fig. S1**. Phylogenetic tree of PhoA, PhoD, PhoX and PafA. **Fig. S2**. Comparison of (a) the number of detected PCGs families and (b) run time in searching against arCOG, COG, eggNOG, KEGG and PCyCDB. **Fig. S3**. Functional composition of PCGs families in seven habitats. **Fig. S4**. Quantitative analysis of transporters in seven habitats. *:*P* < 0.05; **:*P* < 0.01, ***:*P* < 0.001, ****:*P* < 0.0001.

## Data Availability

The PCycDB and utilities are available at https://github.com/ZengJiaxiong/Phosphorus-cycling-database.

## Abbreviations

aa: Amino acids
2AEP: 2-Aminoethylphosphonate
ANOSIM: Analysis of similarity
DOP: Dissolved organic phosphorus
LCA: Lowest common ancestor
MRPP: Multi-response permutational procedure
NMDS: Nonmetric multidimensional scaling
NCycDB: Nitrogen cycling database
NPV: Negative predictive value
ORFs: Open reading frames
PCGs: Phosphorus cycling genes
PCMs: Phosphorus cycling microorganisms
PCycDB: Phosphorus cycling database
PEP: Phosphoenolpyruvate
PGA: Phosphoglycerate
Pi: Inorganic phosphorus
Po: Organic phosphorus
PRPP: α-D-ribose-1-diphosphate-5P
PPV: Positive predictive value
PSB: P solubilizing bacteria
sp.: Species
SCycDB: Sulfur cycling database
SRA: Sequence read archive
WWTP: Wastewater treatment plant

## References

[1] Dai Z, Liu G, Chen H, Chen C, Wang J, Ai S, Wei D, Li D, Ma B, Tang C (2020). Long-term nutrient inputs shift soil microbial functional profiles of phosphorus cycling in diverse agroecosystems. ISME J.

[2] Yang X, Post WM (2011). Phosphorus transformations as a function of pedogenesis: a synthesis of soil phosphorus data using Hedley fractionation method. Biogeosciences.

[3] Walker T, Syers JK (1976). The fate of phosphorus during pedogenesis. Geoderma.

[4] Alori ET, Glick BR, Babalola OO (2017). Microbial phosphorus solubilization and its potential for use in sustainable agriculture. Front Microbiol.

[5] Liang JL, Liu J, Jia P, Yang TT, Zeng QW, Zhang SC, Liao B, Shu WS, Li JT (2020). Novel phosphate-solubilizing bacteria enhance soil phosphorus cycling following ecological restoration of land degraded by mining. ISME J.

[6] Richardson AE, Simpson RJ (2011). Soil microorganisms mediating phosphorus availability update on microbial phosphorus. Plant Physiol.

[7] Bergkemper F, Schöler A, Engel M, Lang F, Krüger J, Schloter M, Schulz S (2016). Phosphorus depletion in forest soils shapes bacterial communities towards phosphorus recycling systems. Environ Microbiol.

[8] McCloskey D, Xu S, Sandberg TE, Brunk E, Hefner Y, Szubin R, Feist AM, Palsson BO (2018). Adaptation to the coupling of glycolysis to toxic methylglyoxal production in tpiA deletion strains of Escherichia coli requires synchronized and counterintuitive genetic changes. Metab Eng.

[9] White DW, Elliott SR, Odean E, Bemis LT, Tischler AD (2018). Mycobacterium tuberculosis Pst/SenX3-RegX3 regulates membrane vesicle production independently of ESX-5 activity. mBio.

[10] Martinez A, Tyson GW, DeLong EF (2010). Widespread known and novel phosphonate utilization pathways in marine bacteria revealed by functional screening and metagenomic analyses. Environ Microbiol.

[11] McGrath JW, Chin JP, Quinn JP (2013). Organophosphonates revealed: new insights into the microbial metabolism of ancient molecules. Nat Rev Microbiol.

[12] Zangelmi E, Stanković T, Malatesta M, Acquotti D, Pallitsch K, Peracchi A (2021). Discovery of a new, recurrent enzyme in bacterial phosphonate degradation:(R)-1-hydroxy-2-aminoethylphosphonate ammonia-lyase. Biochemistry.

[13] Chin JP, Quinn JP, McGrath JW (2018). Phosphate insensitive aminophosphonate mineralisation within oceanic nutrient cycles. ISME J.

[14] Murphy AR, Scanlan DJ, Chen Y, Adams NB, Cadman WA, Bottrill A, Bending G, Hammond JP, Hitchcock A, Wellington EM (2021). Transporter characterisation reveals aminoethylphosphonate mineralisation as a key step in the marine phosphorus redox cycle. Nat Commun.

[15] Hove-Jensen B, Andersen KR, Kilstrup M, Martinussen J, Switzer RL, Willemoës M (2017). Phosphoribosyl diphosphate (PRPP): biosynthesis, enzymology, utilization, and metabolic significance. Microbiol Mol Biol Rev.

[16] Chen X, Jiang N, Condron LM, Dunfield KE, Chen Z, Wang J, Chen L (2019). Impact of long-term phosphorus fertilizer inputs on bacterial phoD gene community in a maize field, Northeast China. Sci Total Environ.

[17] Rasul M, Yasmin S, Suleman M, Zaheer A, Reitz T, Tarkka MT, Islam E, Mirza MS (2019). Glucose dehydrogenase gene containing phosphobacteria for biofortification of Phosphorus with growth promotion of rice. Microbiol Res.

[18] Sebastian M, Ammerman JW (2009). The alkaline phosphatase PhoX is more widely distributed in marine bacteria than the classical PhoA. ISME J.

[19] Steen AD, Crits-Christoph A, Carini P, DeAngelis KM, Fierer N, Lloyd KG, Thrash JC (2019). High proportions of bacteria and archaea across most biomes remain uncultured. ISME J.

[20] Brown CT, Hug LA, Thomas BC, Sharon I, Castelle CJ, Singh A, Wilkins MJ, Wrighton KC, Williams KH, Banfield JF (2015). Unusual biology across a group comprising more than 15% of domain Bacteria. Nature.

[21] Li J, Peng Y, Zhang L, Liu J, Wang X, Gao R, Pang L, Zhou Y (2019). Quantify the contribution of anammox for enhanced nitrogen removal through metagenomic analysis and mass balance in an anoxic moving bed biofilm reactor. Water Res.

[22] Yang X, Chen Y, Guo F, Liu X, Su X, He Q (2020). Metagenomic analysis of the biotoxicity of titanium dioxide nanoparticles to microbial nitrogen transformation in constructed wetlands. J Hazard Mater.

[23] Coutinho FH, Cabello-Yeves PJ, Gonzalez-Serrano R, Rosselli R, Lopez-Perez M, Zemskaya T, Zakharenko A, Ivanov V, Rodriguez-Valera F (2020). New viral biogeochemical roles revealed through metagenomic analysis of Lake Baikal. Microbiome.

[24] Ruvindy R, White RA, Neilan BA, Burns BP (2016). Unravelling core microbial metabolisms in the hypersaline microbial mats of Shark Bay using high-throughput metagenomics. ISME J.

[25] Duhamel S, Diaz JM, Adams JC, Djaoudi K, Steck V, Waggoner EM (2021). Phosphorus as an integral component of global marine biogeochemistry. Nat Geosci.

[26] Tu Q, Lin L, Cheng L, Deng Y, He Z (2019). NCycDB: a curated integrative database for fast and accurate metagenomic profiling of nitrogen cycling genes. Bioinformatics.

[27] Zhang AN, Li L-G, Ma L, Gillings MR, Tiedje JM, Zhang T (2018). Conserved phylogenetic distribution and limited antibiotic resistance of class 1 integrons revealed by assessing the bacterial genome and plasmid collection. Microbiome.

[28] Yu X, Zhou J, Song W, Xu M, He Q, Peng Y, Tian Y, Wang C, Shu L, Wang S (2021). SCycDB: A curated functional gene database for metagenomic profiling of sulphur cycling pathways. Mol Ecol Resour.

[29] Liu Y-Y, Wang Y, Walsh TR, Yi L-X, Zhang R, Spencer J, Doi Y, Tian G, Dong B, Huang X (2016). Emergence of plasmid-mediated colistin resistance mechanism MCR-1 in animals and human beings in China: a microbiological and molecular biological study. Lancet Infect Dis.

[30] Kraus EA, Nothaft D, Stamps BW, Rempfert KR, Ellison ET, Matter JM, Templeton AS, Boyd ES, Spear JR (2020). Molecular evidence for an active microbial methane cycle in subsurface serpentinite-hosted groundwaters in the Samail ophiolite, Oman. Appl Environ Microbiol.

[31] Galperin MY, Makarova KS, Wolf YI, Koonin EV (2015). Expanded microbial genome coverage and improved protein family annotation in the COG database. Nucleic Acids Res.

[32] Kanehisa M, Sato Y, Furumichi M, Morishima K, Tanabe M (2019). New approach for understanding genome variations in KEGG. Nucleic Acids Res.

[33] Huerta-Cepas J, Szklarczyk D, Forslund K, Cook H, Heller D, Walter MC, Rattei T, Mende DR, Sunagawa S, Kuhn M (2016). eggNOG 4.5: a hierarchical orthology framework with improved functional annotations for eukaryotic, prokaryotic and viral sequences. Nucleic Acids Res.

[34] Overbeek R, Begley T, Butler RM, Choudhuri JV, Chuang H-Y, Cohoon M, de Crécy-Lagard V, Diaz N, Disz T, Edwards R (2005). The subsystems approach to genome annotation and its use in the project to annotate 1000 genomes. Nucleic Acids Res.

[35] Wolf YI, Makarova KS, Yutin N, Koonin EV (2012). Updated clusters of orthologous genes for Archaea: a complex ancestor of the Archaea and the byways of horizontal gene transfer. Biol Direct.

[36] Lidbury ID, Fraser T, Murphy AR, Scanlan DJ, Bending GD, Jones AM, Moore JD, Goodall A, Tibbett M, Hammond JP (2017). The ‘known’genetic potential for microbial communities to degrade organic phosphorus is reduced in low-pH soils. MicrobiologyOpen.

[37] Luo H, Lin X, Li L, Lin L, Zhang C, Lin S (2017). Transcriptomic and physiological analyses of the dinoflagellate Karenia mikimotoi reveal non-alkaline phosphatase-based molecular machinery of ATP utilisation. Environ Microbiol.

[38] Gebhard S, Cook GM (2008). Differential regulation of high-affinity phosphate transport systems of Mycobacterium smegmatis: identification of PhnF, a repressor of the phnDCE operon. J Bacteriol.

[39] Thaller MC, Berlutti F, Schippa S, Lombardi G, Rossolini GM (1994). Characterization and sequence of PhoC, the principal phosphate-irrepressible acid phosphatase of Morganella morganii. Microbiology.

[40] Sola-Landa A, Moura R, Martin J (2003). The two-component PhoR-PhoP system controls both primary metabolism and secondary metabolite biosynthesis in Streptomyces lividans. Proc Natl Acad Sci.

[41] Glover RT, Kriakov J, Garforth SJ, Baughn AD, Jacobs WR (2007). The two-component regulatory system senX3-regX3 regulates phosphate-dependent gene expression in Mycobacterium smegmatis. J Bacteriol.

[42] diSioudi B, Grimsley JK, Lai K, Wild JR (1999). Modification of near active site residues in organophosphorus hydrolase reduces metal stoichiometry and alters substrate specificity. Biochemistry.

[43] Quinn JP, Kulakova AN, Cooley NA, McGrath JW (2007). New ways to break an old bond: the bacterial carbon–phosphorus hydrolases and their role in biogeochemical phosphorus cycling. Environ Microbiol.

[44] Stasi R, Neves HI, Spira B (2019). Phosphate uptake by the phosphonate transport system PhnCDE. BMC Microbiol.

[45] Lidbury ID, Borsetto C, Murphy AR, Bottrill A, Jones AM, Bending GD, Hammond JP, Chen Y, Wellington EM, Scanlan DJ (2021). Niche-adaptation in plant-associated Bacteroidetes favours specialisation in organic phosphorus mineralisation. ISME J.

[46] Lidbury ID, Scanlan DJ, Murphy AR, Christie-Oleza JA, Aguilo-Ferretjans MM, Hitchcock A, et al. A widely distributed phosphate-insensitive phosphatase presents a route for rapid organophosphorus remineralization in the biosphere. Proc Natl Acad Sci. 2022;119(5):e2118122119. 10.1073/pnas.2118122119 PMC8812569 35082153

[47] Consortium TU (2021). UniProt: the universal protein knowledgebase in 2021. Nucleic Acids Res.

[48] Edgar RC (2010). Search and clustering orders of magnitude faster than BLAST. Bioinformatics.

[49] Fu L, Niu B, Zhu Z, Wu S, Li W (2012). CD-HIT: accelerated for clustering the next-generation sequencing data. Bioinformatics.

[50] Pal C, Bengtsson-Palme J, Rensing C, Kristiansson E, Larsson DJ (2014). BacMet: antibacterial biocide and metal resistance genes database. Nucleic Acids Res.

[51] Joshi N, Fass J (2011). Sickle: a sliding-window, adaptive, quality-based trimming tool for FastQ files.

[52] Li DH, Luo RB, Liu CM, Leung CM, Ting HF, Sadakane K, Yamashita H, Lam TW (2016). MEGAHIT v1.0: a fast and scalable metagenome assembler driven by advanced methodologies and community practices. Methods.

[53] Hyatt D, Chen G-L, LoCascio PF, Land ML, Larimer FW, Hauser LJ (2010). Prodigal: prokaryotic gene recognition and translation initiation site identification. BMC Bioinformatics.

[54] Ma L, Xia Y, Li B, Yang Y, Li L-G, Tiedje JM, Zhang T (2016). Metagenomic assembly reveals hosts of antibiotic resistance genes and the shared resistome in pig, chicken, and human feces. Environ Sci Technol.

[55] Su J-Q, An X-L, Li B, Chen Q-L, Gillings MR, Chen H, Zhang T, Zhu Y-G (2017). Metagenomics of urban sewage identifies an extensively shared antibiotic resistome in China. Microbiome.

[56] Alcock BP, Raphenya AR, Lau TT, Tsang KK, Bouchard M, Edalatmand A, Huynh W (2020). Nguyen A-LV, Cheng AA, Liu S: CARD 2020: antibiotic resistome surveillance with the comprehensive antibiotic resistance database. Nucleic Acids Res.

[57] Yuan L, Wang Y, Zhang L, Palomo A, Zhou J, Smets BF, Bürgmann H, Ju F (2021). Pathogenic and indigenous denitrifying bacteria are transcriptionally active and key multi-antibiotic-resistant players in wastewater treatment plants. Environ Sci Technol.

[58] Arango-Argoty G, Garner E, Pruden A, Heath LS, Vikesland P, Zhang L (2018). DeepARG: a deep learning approach for predicting antibiotic resistance genes from metagenomic data. Microbiome.

[59] Jahan M, Mahallati MN, Amiri MB, Ehyayi H (2013). Radiation absorption and use efficiency of sesame as affected by biofertilizers inoculation in a low input cropping system. Ind Crop Prod.

[60] Luecke H, Quiocho FA (1990). High specificity of a phosphate transport protein determined by hydrogen bonds. Nature.

[61] Seweryn P, Van LB, Kjeldgaard M, Russo CJ, Passmore LA, Hove-Jensen B, Jochimsen B, Brodersen DE (2015). Structural insights into the bacterial carbon–phosphorus lyase machinery. Nature.

[62] Kim AD, Baker AS, Dunaway-Mariano D, Metcalf W, Wanner B, Martin BM (2002). The 2-aminoethylphosphonate-specific transaminase of the 2-aminoethylphosphonate degradation pathway. J Bacteriol.

[63] Eder S, Shi L, Jensen K, Yamane K, Hulett FM (1996). A Bacillus subtilis secreted phosphodiesterase/alkaline phosphatase is the product of a Pho regulon gene, phoD. Microbiology.

[64] Fatima F, Ahmad M, Verma S, Pathak N. Relevance of phosphate solubilizing microbes in sustainable crop production: a review. Int J Environ Sci Technol. 2021. 10.1007/s13762-021-03425-9.

[65] Li Jt L, Jl WH, Fang Z, Wang X, Feng S, Wang Z, et al. A comprehensive synthesis unveils the mysteries of phosphate-solubilizing microbes. Biol Rev. 2021;96(6):2771-93. 10.1111/brv.12779 PMC9291587 34288351

[66] Reef R, Feller IC, Lovelock CE (2010). Nutrition of mangroves. Tree Physiol.

[67] Hsieh Y-J, Wanner BL (2010). Global regulation by the seven-component Pi signaling system. Curr Opin Microbiol.

[68] Elser J, Bennett E (2011). A broken biogeochemical cycle. Nature.

[69] Udaondo Z, Duque E, Daddaoua A, Caselles C, Roca A, Pizarro-Tobias P, Ramos JL (2020). Developing robust protein analysis profiles to identify bacterial acid phosphatases in genomes and metagenomic libraries. Environ Microbiol.

