# Supplementary material for: PCycDB: a comprehensive and accurate database for fast analysis of phosphorus cycling genes
Source: Microbiome. 2022 Jul 4;10:101. doi: 10.1186/s40168-022-01292-1 (PMC9252087; doi:10.1186/s40168-022-01292-1)
Supplement: Supplementary file 5 — Additional file 4: Fig. S1. Phylogenetic tree of PhoA, PhoD, PhoX and PafA. Fig. S2. Comparison of (a) the number of detected PCGs families and (b) run time in searching against arCOG, COG, eggNOG, KEGG and PCyCDB. Fig. S3. Functional composition of PCGs families in seven habitats. Fig. S4. Quantitative analysis of transporters in seven habitats. *:P < 0.05; **:P < 0.01, ***:P < 0.001, ****:P < 0.0001. [file 40168_2022_1292_MOESM4_ESM.pdf]

Supplementary figures

PCycDB: a comprehensive and accurate database for fast analysis of phosphorus cycling genes

Jiaxiong Zeng[1], Qichao Tu[2], Xiaoli Yu[1], Lu Qian[1], Cheng Wang[1], Longfei Shu[1], Fei Liu[1], Shengwei Liu[1], Zhijian Huang[1], Jianguo He[1], Qingyun Yan[1*], Zhili He[1, 3, *]

[1] Environmental Microbiomics Research Center, School of Environmental Science and Engineering, Southern Marine Science and Engineering Guangdong Laboratory (Zhuhai), State Key Laboratory of Biocontrol, Sun Yat-sen University, Guangzhou 510006, China
[2] Institute of Marine Science and Technology, Shandong University, Qingdao, China
[3] College of Agronomy, Hunan Agricultural University, Changsha 410128, China

[*] To whom correspondence should be addressed.
E-mail: hezhili@mail.sysu.edu.cn; yanqy6@mail.sysu.edu.cn

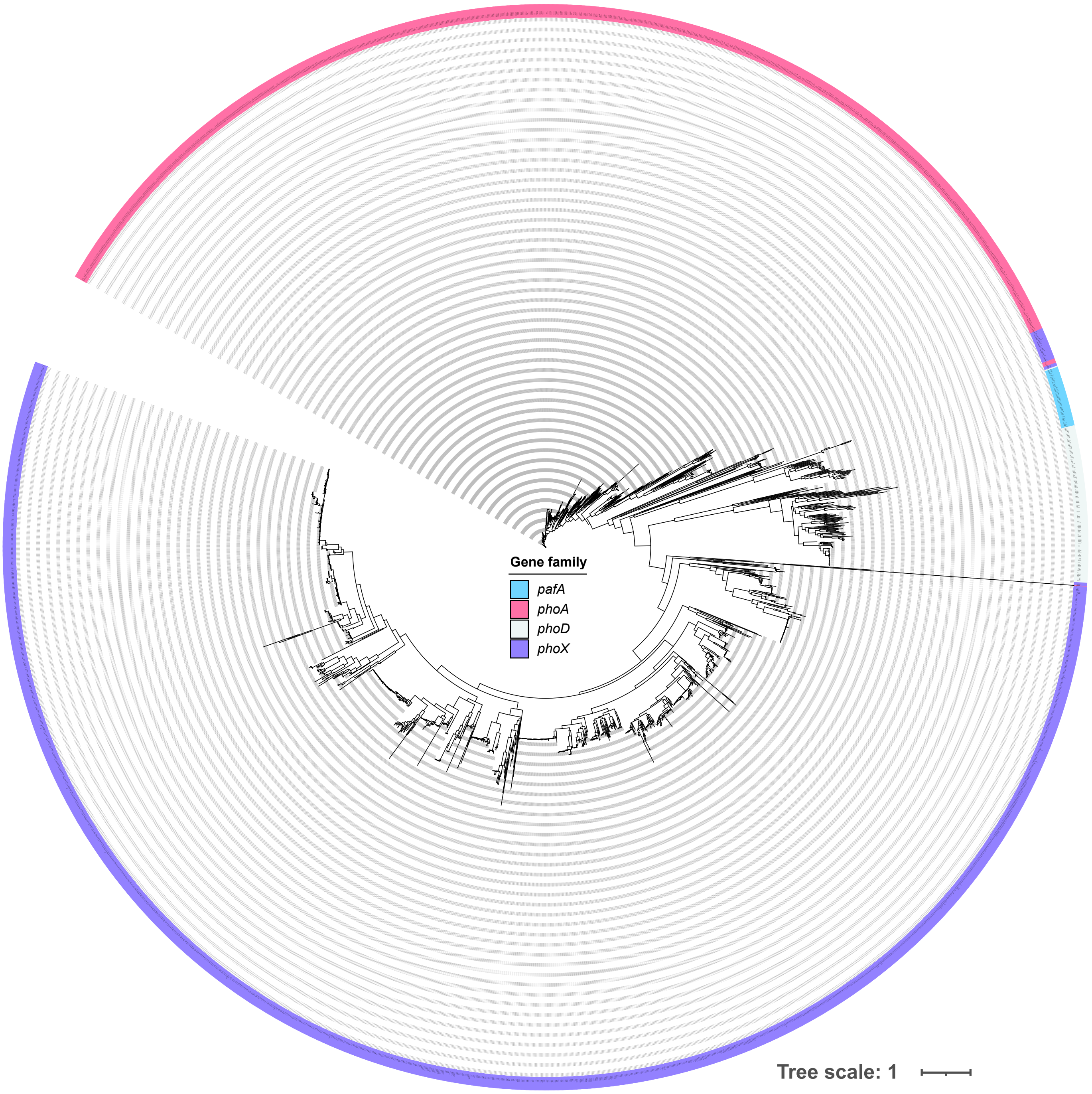

Fig. S1. Phylogenetic tree of PhoA, PhoD, PhoX and PafA.

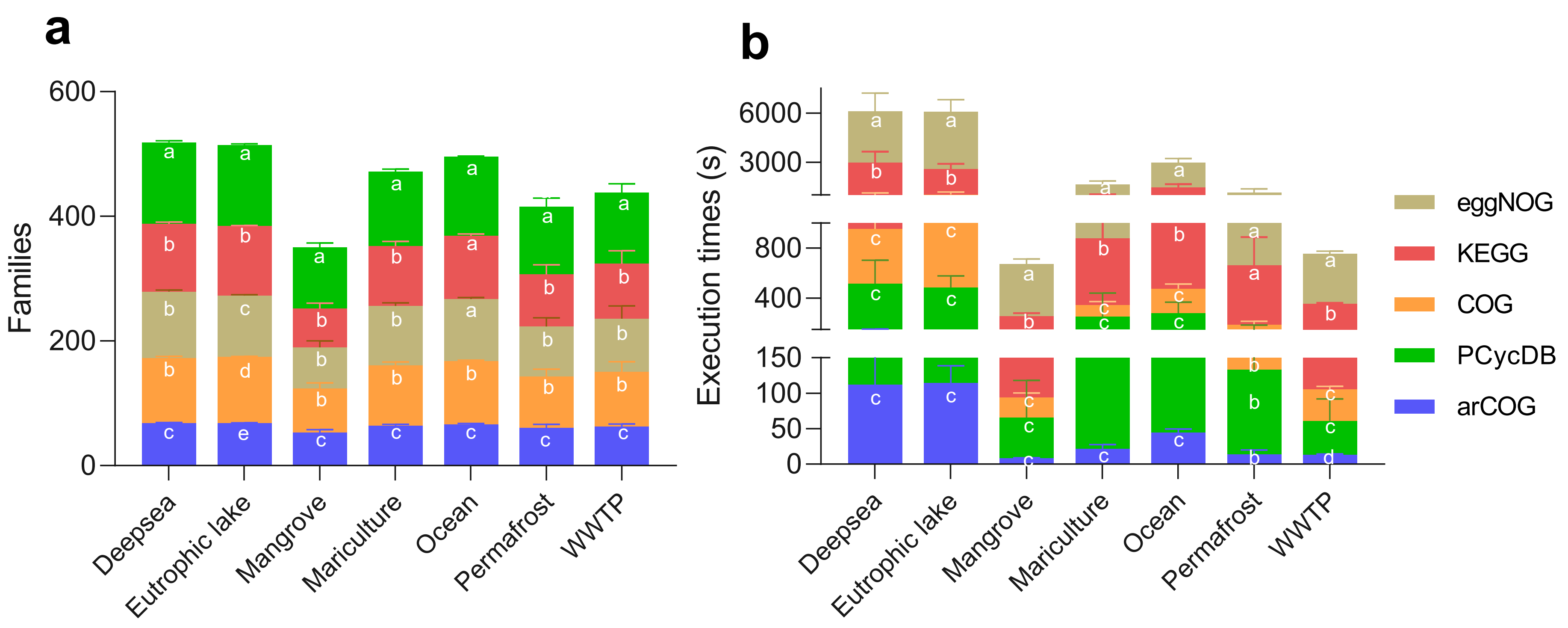

Fig. S2. Comparison of (a) the number of detected PCGs families and (b) run time in searching against arCOG, COG, eggNOG, KEGG and PCyCDB.

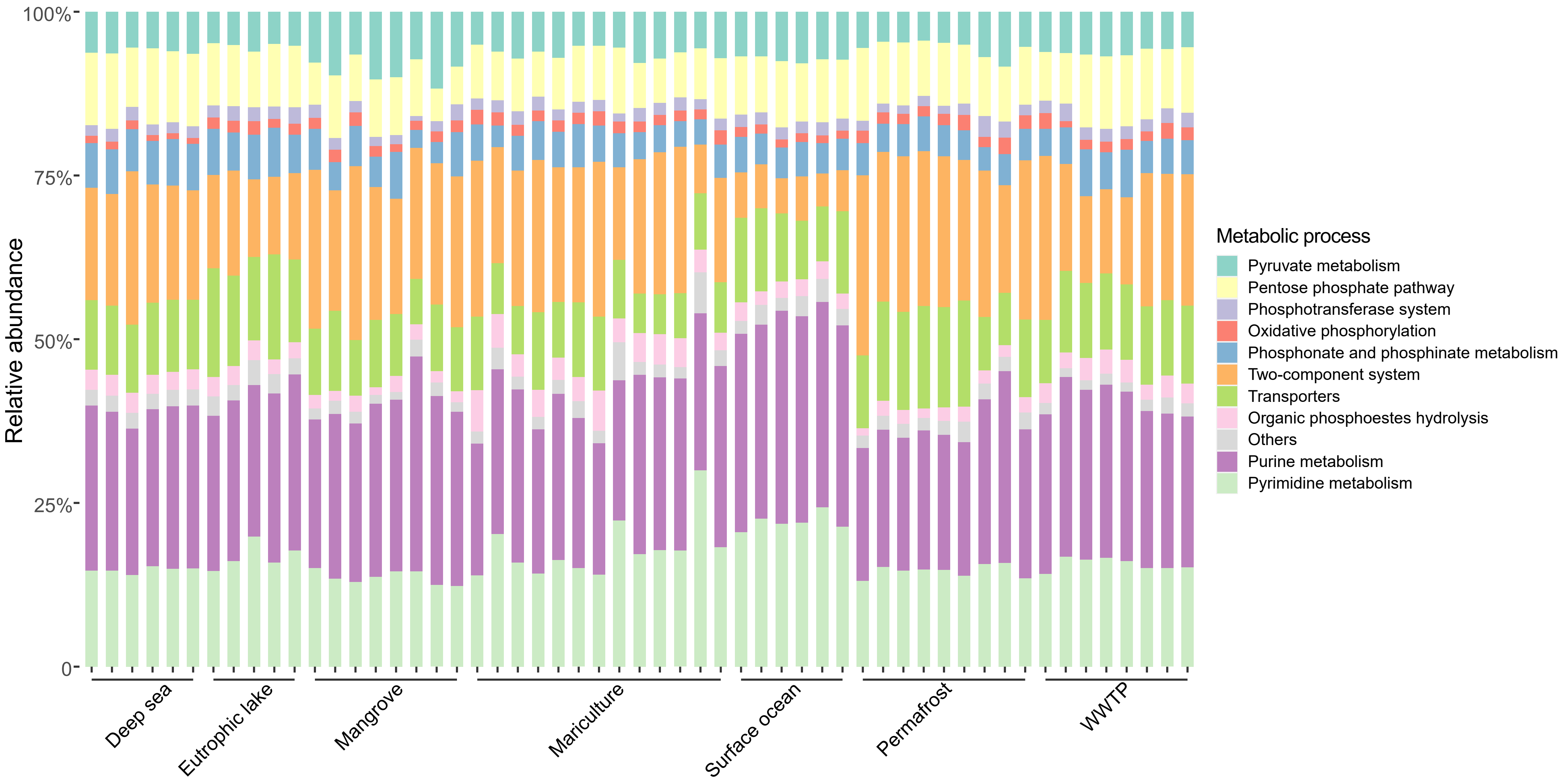

Fig. S3. Functional composition of PCGs families in seven habitats.

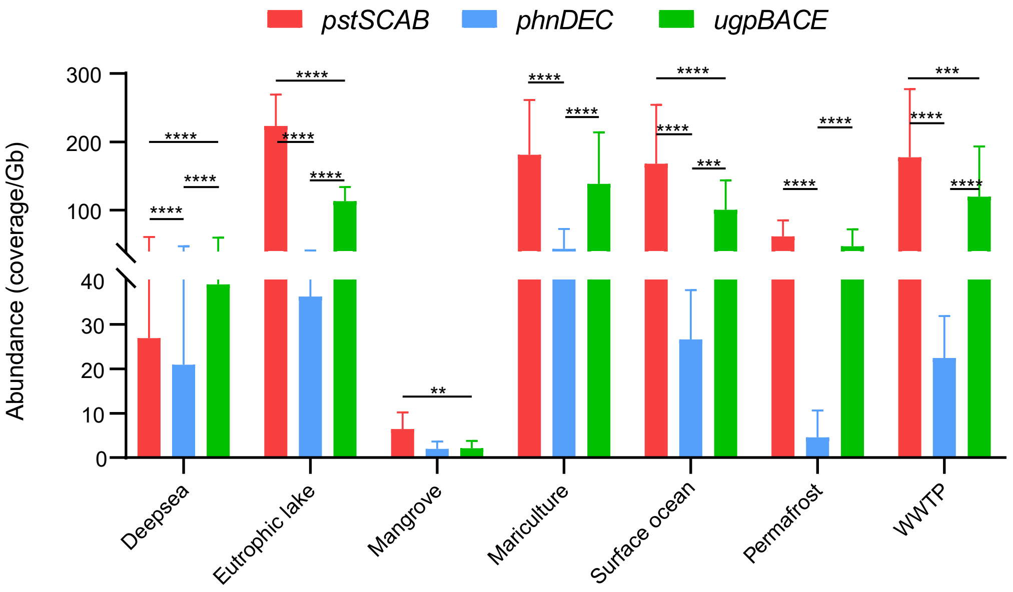

Fig. S4. Quantitative analysis of transporters in seven habitats. *:$P < 0.05$; **:$P < 0.01$, ***:$P < 0.001$, ****:$P < 0.0001$.
